# Inflammatory Th17 cells are correlated with insulin resistance and erythrocyte parameters in overweight and obese children

**DOI:** 10.3389/fendo.2024.1456203

**Published:** 2024-11-14

**Authors:** Dorota Artemniak-Wojtowicz, Małgorzata Rumińska, Anna Stelmaszczyk-Emmel, Monika Paluchowska, Beata Ewa Pyrżak, Anna Małgorzata Kucharska

**Affiliations:** ^1^ Department of Pediatrics and Endocrinology, Medical University of Warsaw, Warsaw, Poland; ^2^ Department of Laboratory Diagnostics and Clinical Immunology of Developmental Age, Faculty of Medicine, Medical University of Warsaw, Warsaw, Poland

**Keywords:** erythrocyte, Th17 cells, insulin resistance, inflammation, obesity, children

## Abstract

**Introduction:**

Obesity is thought to be accompanied by chronic, low-grade, inflammation. The adipocytes are present in the subcutaneous and visceral fat tissue and contribute to the bone marrow cell compartment. Therefore, it poses a question whether the factors influencing adipocyte functions also have an impact on the hematopoietic function of the bone marrow. The aim of our study was to evaluate the association between erythrocyte parameters, the proinflammatory Th17 lymphocytes, and IR markers in children with excessive body weight.

**Methods:**

A total of 27 overweight/obese and 15 normal-weight children aged 8–18 years were enrolled in the study. The analysis included anthropometric measurements, evaluation of Th17 cell frequency, erythrocyte parameters, and carbohydrate metabolism parameters.

**Results:**

In overweight/obese children, erythrocyte count (*p* = 0.00002), hemoglobin (HGB) concentration (*p* = 0.005), and frequency of Th17 cells (*p* = 0.048) were higher. Anthropometric parameters correlated with erythrocyte parameters, as well as Th17 cell frequency in all children. The erythrocyte count correlated with the Th17 subset (*p* = 0.01, *r* = 0.38). Moreover, in all children, the correlation between erythrocyte and fasting insulin (FI) (*p* = 0.00005, *r* = 0.58), HOMA-IR (*p* = 0.00005, *r* = 0.58), and QUICKI (*p* = 0.000042, *r* = −0.59), as well as between HGB and FI (*p* = 0.037, *r* = 0.32), HOMA-IR (*p* = 0.37, *r* = 0.32), and QUICKI (*p* = 0.049, *r* = −0.31) was found. In the overweight/obese group, erythrocyte count correlated with insulin 2 h after the oral glucose tolerance test (OGTT) (*p* = 0.04, *r* = 0.4), while HGB correlated with glucose and insulin 2 h after OGTT (*p* = 0.018, *r* = 0.45; *p* = 0.04, *r* = 0.44, respectively).

**Conclusions:**

Our study confirmed that the erythrocyte parameters are higher in children with obesity and are positively correlated with insulin resistance and proinflammatory Th17 lymphocyte. Thus, it can be concluded that erythrocyte parameters reflect the risk of developing IR in response to chronic inflammation associated with obesity. These are simple, easily accessible, and repeatable tests that, in the assessment of obese patients, may herald the developing metabolic syndrome and serve as a helpful additional tool for assessing the effectiveness of treatment.

## Introduction

Obesity is one of the most common chronic diseases worldwide. In the pediatric population, its incidence has increased dramatically from 4% in 1975 to 18% in 2016 (1). In the last decades, it has been well established that obesity is accompanied by generalized, chronic, low-grade sterile inflammation. This condition alters the peripheral T cells’ profile, increasing Th17 cells, which, in turn, facilitates proinflammatory milieu formation (2, 3) and alters numerous functions of the target cell. The adipocytes are cells directly dependent on the amount of fat tissue. They not only are present in the subcutaneous or visceral fat tissue, but also contribute to bone marrow cells’ compartment. Therefore, it poses a question whether the factors influencing adipocyte functions also have an impact on the hematopoietic function of the bone marrow. Several articles have shown that obesity was associated with increased erythropoiesis. Christakoudi et al. (4) in the UK Biobank cohort observed that in obesity uncomplicated with diabetes, a larger body mass index (BMI) and allometric body shape index (ABSI) are associated with increased erythropoiesis and reticulocyte immaturity (4). Furthermore, Cheng et al. (5) have shown in a systematic review that obese patients tended to have higher hemoglobin (HGB) and ferritin concentrations compared to the non-obese patients (5). The mechanisms involved in the regulation of erythropoiesis in obesity are not fully understood. It could be dependent on many factors, including obesity-induced inflammation and insulin hypersecretion (6). Bersch et al. (7) reported that the stimulatory effect of subnanomolar concentrations of insulin on erythropoiesis *in vitro* suggests that insulin can also modulate erythropoiesis *in vivo* (7). The relationships between insulin resistance (IR) and erythrocyte parameters in adults have been evaluated in several studies (8–11). Ellinger et al. (12) analyzed laboratorial exams from 925 subjects and found that HOMA (homeostasis model assessment) correlated positively with erythrocyte count and HGB concentration, among other factors. Furthermore, subjects in the upper quartile of IR had higher levels of HGB and erythrocyte count than those in the lower quartile (12). Furthermore, Choi et al. (13), in a cross-sectional study involving 1,314 people without diabetes over the age of 60, found that HGB concentration was associated with HOMA and other factors such as BMI or fasting plasma insulin level (13). However, there are still relatively few observations regarding this issue in children with obesity.

In our previous study, we found that obese children had a higher erythrocyte count (14). We have hypothesized that it may relate to the inflammatory process or IR. Therefore, the aim of the present study was to evaluate the association between erythrocyte parameters, the proinflammatory Th17 lymphocytes, and IR markers in children with excessive body weight.

## Methods

This study was conducted in the Department of Pediatrics and Endocrinology at the Medical University of Warsaw. A study group consisted of 27 overweight/obese children (12 girls and 15 boys) aged 8–18 years recruited among children hospitalized in the clinic due to excessive body weight between August 2016 and October 2020.

Children with secondary overweight/obesity due to hormonal, central nervous system disorders, or genetic diseases as well as allergy, hematological, or chronic or acute illness were excluded from the study. Additionally, participants who had suffered from inflammatory diseases or taken medications such as antibiotics or glucocorticoids, which could affect serum inflammatory markers, within the last 3 months were also excluded.

The control group consisted of 15 normal-weight and healthy children (7 girls and 8 boys), who were age- and sex-matched.

The protocol of the study was approved by the Bioethics Committee at the Medical University of Warsaw (No. KB/61/2016, KB/52/A/2016).

All parents and patients over the age of 16 gave informed consent before they participated in the study.

The analysis included anthropometric measurements, taken by one anthropologist. Height (cm), body weight (kg), waist circumference (WC) (cm), and hip circumference (HC) (cm) were measured according to the WHO recommendations (15). These parameters were normalized for calendar age according to the national growth charts for children aged 3–18 years (16). The waist-to-hip ratio (WHR) and the waist-to-height ratio (WHtR) were calculated. The BMI was calculated by dividing the weight (kg) by the square of the height in meters (m^2^). Obesity was defined by BMI >+2 standard deviation score (SDS), and overweight was defined as BMI between +1 and +1.9 SDS. The control group consisted of children with normal somatic parameters (−1 < BMI < +1 SDS).

Blood samples collected in EDTA were measured by standard methods (Sysmex XN 1000i hematological analyzer) in the morning after an overnight fasting period. The analysis included erythrocyte count, HGB concentration, values of mean corpuscular volume (MCV), mean corpuscular hemoglobin (MCH), mean corpuscular hemoglobin concentration (MCHC), and RDW (red blood cell distribution width).

The method of Th17 cell evaluation was described in our previous paper (17). Th17 cells were assessed by flow cytometry as follows (in short): 50 μL of fresh venous blood after staining with monoclonal antibodies (according to the manufacturer’s instructions—Becton Dickinson Biosciences)—anti-CD3 APC-H7, anti-CD4 PE-Cy7, and anti-CD196 APC (CCR6) (Becton Dickinson, Franklin Lakes, NJ, USA)—was washed [in a washing buffer (0.9% NaCl)], permeabilized [IntraPrep Permeabilization Reagent 1 and 2 (Immuno-tech SAS, Beckman Coulter Company, 13276 Marseille Cedex 9, France)], and stained intracellularly with anti-IL-17A PE monoclonal antibody. Samples were stored at room temperature before analysis. Appropriate isotype-matched controls were used in the staining procedures. Flow cytometry was performed on a FACS Canto II flow cytometer (Becton Dickinson, Franklin Lakes, NJ, USA) using BD FACS Diva 8.0.1 software. Gates were preset, and the measurements were performed blinded for sample identity. Th17 cells were defined as CD3^+^CD4^+^CD196^+^ expressing intracellular IL-17A (CD3^+^CD4^+^CD196^+^IL-17^ic+^). The value of Th17 cells was expressed as a percentage of total CD3^+^CD4^+^ Th cells. A representative flow cytometry chart of flanking of Th 17 cells in an obese child can be found in the **Supplementary Materials**.

Biochemical laboratory tests in obese and overweight children included the oral glucose tolerance test (OGTT) (1.75 g of glucose/kg body weight, no more than 75 g) with insulin and glycated hemoglobin A1c value (HbA1c). Blood tests in the control group included fasting glucose (FG) (mg/dL) and fasting insulin (FI) (μIU/mL) concentrations and HbA1c (%). HOMA-IR (homeostasis model assessment insulin resistance index) and QUICKI (quantitative insulin sensitivity check index) have been established as indicators of IR. HOMA-IR was calculated as follows: HOMA-IR = [glucose (mmol/l) × insulin (μIU/mL)]/22.5, glucose conversion factor: mmol/L = mg/dL × 0.05551. QUICKI was calculated as follows: QUICKI = 1/[log insulin (μIU/mL) + log glucose (mg/dL).

Statistical analysis was performed using the SPPS 13.3 software. The Shapiro–Wilk test was used to check the normality of the data distribution. Data were presented as means and standard deviation (SD) for normally distributed data or median with interquartile range (IQR), when the distribution was different from normal. A Student’s *t*-test (parametric data) or Mann–Whitney *U* test (nonparametric data) was performed to compare the data of overweight/obese children and the control group. Relationship analyses were performed using the Spearman’s rank correlation coefficient test. A *p*-value < 0.05 was considered as statistically significant.

## Results

The study group characteristics are shown in **Table 1**.

**Table 1:** Clinical characteristics of participants.

Variable	Study group	Control group	*p*-value
Number	27	15	
Sex (M/F)	15:12 (56:44%)	8:7 (54:46%)	ns
Age	12.67 ± 2.55	12.65 ± 2.64	ns
BMI (kg/m2)	29.6 (27.2-33.7)	18.6 (16.5-20.2)	<0.001
BMI SDS	2.3 (1.9–2.5)	0.1 (-0.5–0.3)	<0.001
WC (cm)	93.24 ± 14.23	63.91 ± 3.36	<0.001
WC SDS	3.7 (2.6–4.4)	−0.3 (−0.7–0.1)	<0.001
HC (cm)	105.00 (94.00–109.00)	80.00 (74.00–87.00)	<0.001
WHR	0.88 ± 0.055	0.80 ± 0.05	<0.001
WHtR	0.56 (0.53–0.59)	0.42 (0.40–0.44)	<0.001

Data are presented as median values with interquartile range as appropriate or mean ± standard deviation (SD). BMI, body mass index; BMI SDS, body mass index standard deviation score; WC, waist circumference; WC SDS, waist circumference standard deviation score; HC, hip circumference; WHR, waist-to-hip ratio; WHtR, waist-to-height ratio. A *p* < 0.05 was considered significant; ns, non-significant.

A representative flow cytometry analysis of Th 17 cells in an obese child, which was previously presented (17), can be found in the **Supplementary Materials**.

A data summary of erythrocyte parameters, Th17 lymphocyte frequency, and indices of IR in overweight/obese and normal-weight children is presented in **Table 2**.

**Table 2:** A data summary of erythrocyte parameters, Th17 lymphocyte frequency, and indices of insulin resistance in overweight/obese children (study group) and normal-weight children (control group).

Variable	Study group (*n* = 27)	Control group (*n* = 15)	*p*-value
Th17 cells (% of CD^3+^ CD^4+^)	0.097 (0.044–0.289)	0.041 (0.023–0.099)	0.048
Erythrocyte (cells × 10^6^/μL)	5.13 ± 0.37	4.70 ± 0.25	0.00002
HGB (g/dL)	14.0 (13.1–14.7)	13.00 (12.2–13.5)	0.005
MCV (fL)	79.57 ± 4.66	80.57 ± 5.74	ns
MCH (pg)	26.84 ± 2.08	27.19 ± 2.26	ns
MCHC (g/dL)	33.85 ± 0.97	33.74 ± 1.12	ns
RDW (%)	13.20 (12.4–13.7)	12.6 (12.4–14.1)	ns
Fasting glucose (mg/dL)	85.96 ± 7.22	83 ± 5.7	ns
Fasting insulin (μIU/mL)	20.4 (10.1–36.7)	3.92 (2.89–10.6)	0.000015
HbA1c (%)	5.3 (5.15–5.45)	5.3 (5.0–5.4)	ns
HOMA-IR	3.8 (2.09–7.66)	0.79 (0.58–2.2)	0.000012
QUICKI	0.31 ± 0.03	0.39 ± 0.05	<0.0000001

Data are presented as median values with interquartile range as appropriate or mean ± standard deviation (SD); HGB, hemoglobin; MCV, mean corpuscular volume; MCH, mean corpuscular hemoglobin; MCHC, mean corpuscular hemoglobin concentration; RDW, red blood cell distribution width; HbA1c, glycated hemoglobin; HOMA-IR, homeostasis model assessment-insulin resistance; QUICKI, quantitative insulin sensitivity check index. A *p* < 0.05 was considered significant; ns, non-significant.

In the group of all children, erythrocyte count positively correlated with BMI (*p* = 0.000004, *r* = 0.65), BMI SDS (*p* = 0.000002, *r* = 0.66), WC SDS (*p* = 0.000004, *r* = 0.67), WHR (*p* = 0.00001, *r* = 0.67), WHtR (*p* = 0.000001, *r* = 0.69), and HGB concentration with BMI (*p* = 0.004, *r* = 0.43), BMI SDS (*p* = 0.005, *r* = 0.42), WC SDS (*p* = 0.025, *r* = 0.36), WHR (*p* = 0.003, *r* = 0.49), and WHtR (*p* = 0.008, *r* = 0.42), whereas the percentage of Th17 cells was weakly correlated with BMI (*p* = 0.048, *r* = 0.31), BMI SDS (*p* = 0.032, *r* = 0.33), and WHtR (*p* = 0.02, *r* = 0.37).

In the group with obesity, there was a statistically significant positive correlation between erythrocyte and BMI (*p* = 0.02, *r* = 0.43), BMI SDS (*p* = 0.02, *r* = 0.43), WC SDS (*p* = 0.004, *r* = 0.52), WHR (*p* = 0.02, *r* = 0.45), and WHtR (*p* = 0.001, *r* = 0.56), as well as Th17 cells with WHR (*p* = 0.02, *r* = 0.45). A correlation between the main hematological variables of erythropoiesis and the parameters of IR was also found (see **Table 2**).

In all children, the erythrocyte value correlated with FI (*p* = 0.00005, *r* = 0.58), HOMA-IR (*p* = 0.00005, *r* = 0.58), QUICKI (*p* = 0.000042, *r* = -0.59), and HGB concentration with FI (*p* = 0.037, *r* = 0.32), HOMA-IR (*p* = 0.037, *r* = 0.32), and QUICKI (*p* = 0.049, *r* = -0.31). In addition, RDW correlated positively with HbA1c (*p* = 0.006, *r* = 0.45).

Furthermore, in the group with obesity, a significant correlation was found between the erythrocyte and insulin value after 2 h of OGTT (*p* = 0.04, *r* = 0.4), while HGB correlated with glucose and insulin after 2 h of OGTT (*p* = 0.018, *r* = 0.45; *p* = 0.04, *r* = 0.44, respectively). Erythrocyte counts correlated positively with a percentage of proinflammatory Th17 cells (*p* = 0.01, *r* = 0.38).

## Discussion

In our previous study, we found that the percentage of Th17 cells correlated with parameters of carbohydrate metabolism (17). This finding reflects the relationship between obesity-associated inflammation and carbohydrate metabolism. In some studies, it was found that adults and children with obesity have increased erythrocyte count and HGB concentration (3, 18), although some authors have not confirmed these results (19, 20). In our study, children with obesity had a statistically significant higher erythrocyte count and HGB concentration compared to normal-weight children, and these values were positively correlated with anthropometric indices of obesity (including BMI SDS). It seems to be particularly important that there is a statistically significant relationship between the erythrocyte count and abdominal obesity indices (WC SDS, WHR, and WHtR) and this correlation was observed in children with obesity as well as in the group of all children. However, Barazzoni et al. (21) demonstrated in multiple regression analysis that obesity per se is not independently associated with higher hematological parameters, whereas abdominal fat accumulation and IR appear to mediate association between BMI and erythrocyte parameters (21). Previously, Barbieri et al. (10) provided evidence of an association between IR and erythropoiesis in a large group of adults. Similarly, in the Mendelian randomization analysis performed by Nguyen et al. (22), higher FI was correlated with increased HGB, erythrocyte, and reticulocyte counts. These results could be explained by the stimulating effect of hyperinsulinemia on erythropoiesis in obesity. Insulin as an anabolic hormone stimulates the growth of various cell types and promotes the growth of human bone marrow cells as well as circulating erythroid progenitor cells (23, 24). The erythrocyte count and mass greatly affect the rheologic properties of blood and are considered strong independent predictors of major cardiovascular acute events (25). The influence of hyperinsulinemia on erythrocytes could explain the high rates of cardiovascular events that have been found in patients with the IR syndrome (10). In addition, HGB plays a key role in regulating sCD40L levels (26), which has been shown to participate in thrombus formation and inflammation, as an independent risk factor for atherosclerosis and metabolic syndrome (27). Our results are in line with these reports. FI, HOMA-IR, and QUICKI correlated with both the erythrocyte count and HGB in the group of all children. One of the mechanisms responsible for the IR in obesity is obesity-induced low-grade chronic inflammation (28).

In our study, we found that the erythrocyte count correlated positively with the percentage of proinflammatory Th17 lymphocytes, which may suggest that the increased erythrocyte count may be dependent on the obesity-induced inflammation. Mărginean et al. (3) reported that children with excessive body weight had higher erythrocyte count and higher concentration of C-reactive protein compared to normal-weight children. In addition, ferritin, which is considered a marker of inflammation, is higher in obese individuals, which may lead to an increase in erythrocyte count (29). An attractive explanation for the direct relationship between obesity and erythropoiesis seems to be the bone marrow adipose tissue (BMAT) function. It influences bone marrow homeostasis: bone health and hematopoietic stem cell’s (HSC’s) function (30). Bone marrow adipocytes support and help to maintain survival and the development of HSC, which was well documented *in vitro* by Mattiucci et al. (31). Bone marrow adipocytes can produce cytokines regulating hematopoiesis, and additionally, adipokines released by BMAT, such as TNF-α, IL-6, and CCL2/MCP-1, contribute to the chronic low-grade systemic inflammation processes (30). Considering other mechanisms in the HSC homeostasis and hematopoiesis, the influence of a relative hypercortisolemia associated with obesity should not be forgotten, but we did not assess the concentration of cortisol in this study. In addition, obesity-related comorbidities, such as obstructive sleep apnea or obesity hypoventilation syndrome, may lead to secondary polycythemia, but these effects are more common in adults (32).

The limitation of our study is a relatively small group of patients that may have an impact on the statistical power of results. Moreover, our study shows an association, rather than a direct cause-end-effect relationship, between erythrocyte parameters and Th17 cells and IR markers. In the literature, the data on potential interactions between obesity-induced inflammation, IR, and erythrocyte parameters in children and adolescents are limited. It is essential to confirm these findings and explore the underlying mechanisms of this association in children.

## Conclusions

Our study confirmed that the erythrocyte parameters are significantly higher in children with obesity and are positively correlated with IR and obesity-induced inflammation reflected by an increased Th17 lymphocyte percentage. Therefore, it can be concluded that erythrocyte parameters reflect the risk of developing IR in response to chronic inflammation associated with obesity. These are simple, easily accessible, and repeatable tests that, in the assessment of obese patients, may herald the developing metabolic syndrome and serve as a helpful additional tool for assessing the effectiveness of treatment.

Prospective interventional studies are needed to confirm the usefulness of these parameters in predicting the effects of obesity treatment in children.

## Data Availability

The raw data supporting the conclusions of this article will be made available by the authors, without undue reservation.
