# Supplementary figures and images for: Inflammatory Th17 cells are correlated with insulin resistance and erythrocyte parameters in overweight and obese children
Source: Front Endocrinol (Lausanne). 2024 Nov 14;15:1456203. doi: 10.3389/fendo.2024.1456203 (PMC11602275; doi:10.3389/fendo.2024.1456203)

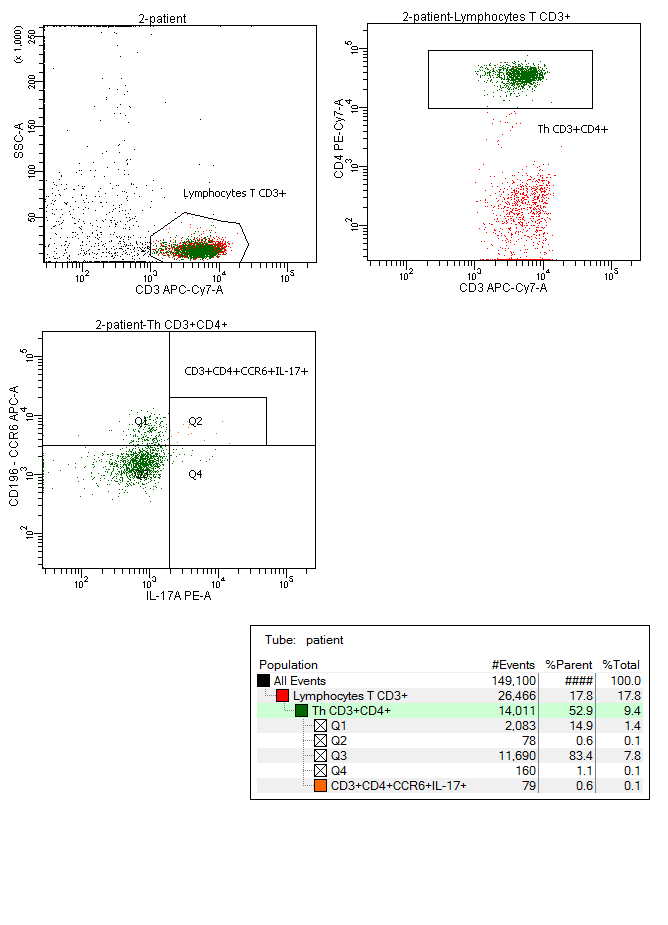


Figure 1. Representative flow cytometry analysis of Th17 cells in obese child.

Supplement: Supplementary file 1 [file DataSheet1.docx]
